# Malnutrition in the Critically Ill Child: The Importance of Enteral Nutrition

**DOI:** 10.3390/ijerph8114353

**Published:** 2011-11-21

**Authors:** Marta Botrán Prieto, Jesús López-Herce Cid

**Affiliations:** 1 Pediatric Intensive Care Department, Hospital General Universitario Gregorio Marañón, No. 47 Doctor Castelo, Madrid 28009, Spain; E-Mail: martabotran@hotmail.com; 2 Servicio de Cuidados Intensivos Pediátricos, Hospital General Universitario Gregorio Marañón, No. 47 Doctor Castelo, Madrid 28009, Spain

**Keywords:** nutrition, malnutrition, enteral nutrition, parenteral nutrition, critically ill children

## Abstract

Malnutrition affects 50% of hospitalized children and 25–70% of the critically ill children. It increases the incidence of complications and mortality. Malnutrition is associated with an altered metabolism of certain substrates, increased metabolism and catabolism depending on the severity of the lesion, and reduced nutrient delivery. The objective should be to administer individualized nutrition to the critically ill child and to be able to adjust the nutrition continuously according to the metabolic changes and evolving nutritional status. It would appear reasonable to start enteral nutrition within the first 24 to 48 hours after admission, when oral feeding is not possible. Parenteral nutrition should only be used when enteral nutrition is contraindicated or is not tolerated. Energy delivery must be individually adjusted to energy expenditure (40–65 kcal/100 calories metabolized/day) with a protein delivery of 2.5–3 g/kg/day. Frequent monitoring of nutritional and metabolic parameters should be performed.

## 1. Introduction

Malnutrition affects millions of children throughout the World. Recently, the humanitarian organization Save the Children warned that “chronic malnutrition affects 178 million children in the World and is the third cause of all childhood deaths worldwide” [1].

Children under 5 years of age in developing countries are particularly vulnerable to malnutrition. However, malnutrition also exists in children in developed countries, in families with few resources or due to disturbances of feeding behavior or diseases that affect eating or the absorption of nutrients.

Infants and children are very susceptible to problems of nutrition. In comparison with adults, children have lower percentages of muscle mass and fat and therefore have fewer reserves and higher resting energy expenditure. For this reason, children have a poorer tolerance to fasting than adults, they are particularly susceptible to protein depletion, and they have an increased risk of developing malnutrition when they suffer serious illness [2]. It must not be forgotten that children are in a growth and development phase, with nutritional requirements that are greater and clearly differentiated from those of adults and that vary according to the stage of growth.

In the ill child, malnutrition is usually of multifactorial origin. It is associated with an altered metabolism of certain substrates, increased or decreased metabolism and catabolism depending on the severity and kind of the lesion, and reduced nutrient delivery. The presence of malnutrition prior to admission worsens the prognosis in the critically ill child and, furthermore, severe illness has marked repercussions on the nutritional status of these patients [3].

Malnutrition interferes with the appropriate response of the body to disease and predisposes to infection and to the onset of multiorgan failure, increasing morbidity and mortality, the mean length of hospital stay, and health costs [2,4–7].

## 2. Incidence of Malnutrition in Hospitalized Children

Malnutrition is common at the time of hospital admission and tends to increase during the hospital stay. In Europe and North America, 40% to 50% of hospitalized patients can be at risk of malnutrition. A number of studies have demonstrated that malnutrition affects 50% of children and adolescents during their hospital admission. The incidence of malnutrition in the critically ill child varies between 25% and 70% depending on the series [2,6–11].

Improvements in the evaluation and monitoring of the nutritional status in the critically ill or chronically ill child, and individualization of nutritional treatment in these patients, appears to have reduced the prevalence of malnutrition in paediatric intensive care units (PICU) to some extent. However, the figures continue to be very high [11–13].

## 3. Types of Malnutrition in Children

Strictly speaking, the term malnutrition refers to any type of alteration of the nutritional status and includes nutritional deficiencies, obesity, and the use of inappropriate diets. However, the term is typically used to refer to nutritional deficiencies.

Nutritional deficiency has traditionally been defined in two ways, depending on the underlying deficit: calorie malnutrition or marasmus and protein malnutrition or kwashiorkor.

Marasmus is caused mainly by a deficit in energy provision and is characterized by a pronounced loss of fat and muscle, leading to emaciation, with the child acquiring a cachectic appearance. Apart from their low weight and height for age, children with energy malnutrition present marked weakness and suffer frequent infections. Other symptoms include dry and wrinkled skin, bradycardia, and bradypnoea.

Kwashiorkor is caused by protein deficiency and is characterized by a loss of appetite, apathy, fluid retention and oedema, alterations of the skin, changes in hair colour, anaemia, and diarrhoea.

However, this clinical classification is subjective and it must also be taken into account that combined clinical states of protein and energy malnutrition are very common. Protein-energy malnutrition, in which there is a combination of energy deficit and, to a lesser degree, protein deficit, is the most important and common form of malnutrition.

Malnutrition can be classified according to its origin as primary, when the cause is a deficit in delivery (inadequate ingestion or gastrointestinal disorders), and secondary, when there is an underlying chronic disease or disorder that causes the malnutrition.

Malnutrition can also be classified as acute or chronic according to the time of onset and duration of the condition. Acute malnutrition occurs when there are recent deficits of supply or the sudden onset of an extensive lesion that leads to high levels of catabolism, whereas chronic malnutrition or growth delay is due to a persistent deficit in nutrient delivery, chronic disease, or acute disorders with a prolonged clinical course [5]. Acute malnutrition mainly affects weight whereas chronic nutrition affects both weight and height in children.

Other forms of malnutrition arise due to specific deficiencies of certain micronutrients, such as the vitamins or minerals. The most common deficiencies in the community are of vitamin A, iron, and iodine, although alterations of magnesium, zinc, and copper are also common in the critically ill child.

## 4. Risk Factors for Malnutrition in the Critically Ill Child

There are numerous factors that contribute to the onset of malnutrition in children admitted to intensive care. The incidence of malnutrition is higher in children under two years of age, in those with a prolonged hospital stay, and in those who require mechanical ventilation. Children with congenital heart disease and extensive burn injuries are also at increased risk of malnutrition [11].

An additional factor is that critically ill children frequently receive an insufficient calorie and protein delivery because enteral or parenteral nutrition cannot be initiated due to gastrointestinal intolerance or the need to restrict fluid intake, initiation is delayed, or there are interruptions in enteral nutrition in order to administer medication or to perform interventions requiring sedation [14].

## 5. Diagnosis of Malnutrition

The diagnosis of malnutrition in the ill child must be based on an objective evaluation of the nutritional status; this includes an adequate history of recent food intake and weight loss, anthropometric measurements, analysis of biochemical parameters and cellular immunity, and calculation of the body composition [8,15]. An adequate nutritional evaluation is essential in order to institute early nutritional intervention [8,11,16,17].

### 5.1. Anthropometric Measurements

Anthropometric evaluation is the simplest method for evaluating the nutritional status of a child. It has the advantages of being applicable to all patients, it is non-invasive, and it is not an expensive technique [16]. Anthropometric measurements in the child are more quantifiable and practical in the outpatient clinic and the World Health Organization therefore recommends the use of the following indices: weight for age, height for age, weight for height, mid-upper arm circumference, and birth weight.

The weight and height of the patient at the time of hospital admission allow us to perform a simple evaluation of the nutritional status and to calculate a number of nutritional indices. Weight is a good parameter for evaluating the effectiveness of nutrition in the child, though it is difficult to measure in the critically ill child on mechanical ventilation and with numerous catheters. Furthermore, it has a low sensitivity in the short term, and can be affected by variations in body composition (oedema or dehydration) without indicating changes in the nutritional status [16,18].

Measurement of the skinfolds is a cheap and relatively easy technique for studying the protein and fat components of the body; this is useful for the evaluation and monitoring of nutritional status [19]. The most widely used parameters are the tricipital skinfold thickness and mid-upper arm circumference. However, the measurement and interpretation of skinfold thickness has significant limitations in critically ill patients, as oedema increases the thickness of the fold, leading to overestimation of the nutritional status. In addition, skinfold parameters have a low sensitivity for short-term changes in the nutritional status.

### 5.2. Haematological Parameters

Haematological parameters are of very little value in the nutritional evaluation of the critically ill child as other factors such as haemorrhage, recent surgery, blood extractions, chronic infection or inflammation, and blood transfusions will alter their values to a much greater degree than the nutritional status [16].

### 5.3. Biochemical Parameters

In contrast, biochemical parameters are useful for evaluating and monitoring a patient’s metabolism during hospital admission. Very low plasma concentrations of cholesterol and lipoproteins have been found in critically ill adults; these changes are due to an interruption of the enterohepatic circulation secondary to fasting or parenteral nutrition, infection, the administration of antibiotics, intestinal bacterial overgrowth, and multiorgan failure [20]. Some studies have found low values of total cholesterol, LDL-cholesterol, HDL-cholesterol, and apolipoprotein B in critically ill adults, with normal apoprotein A-I and triglyceride levels [20]. In children, low cholesterol and triglyceride levels are also common at the time of admission but they increase significantly with enteral nutrition, suggesting good lipid metabolism [16,20–22]. It is also common to detect high triglycerides levels in relation to inflammatory states [23].

Hyperglycaemia is a common finding in critically ill children in the first 48 hours of their admission and is usually due to resistance or a decreased sensitivity of the tissues to insulin [16,17,21,22,24]. However, after this initial period, critically ill children usually show good tolerance to enterally administered carbohydrates [16,21,22]. Blood glucose values are therefore not good indicators of the nutritional status of the critically ill patient.

The Creatinine/Height Index measures global muscle catabolism and its values are affected by the quantity and type of proteins in the diet. In the critically ill patient, the creatinine/height index is able to detect malnutrition on admission, but its value as a variable for monitoring patients is an unresolved issue and also is not useful in renal failure [4,23]. Some studies suggest that a fall in the creatinine/height index could be an indicator of a risk of death, though other studies have not confirmed these findings [16].

The Nitrogen Balance is not a valid parameter for the diagnosis of malnutrition, although it can be used to control nutritional treatment and as a nutritional prognostic indicator (4). However, it must be taken into account that the critically ill patient presents acute phases of catabolism during which there is a negative nitrogen balance. Although the aim of nutrition is to achieve a positive or neutral nitrogen balance through an adequate energy and protein delivery, the hypercatabolic state that develops in the critically ill child as a response mechanism to the aggression cannot be initially blocked despite adequate protein delivery. At later stages a positive nitrogen balance can be reached with an adequate nutrition delivery [7,16,21,23].

Certain serum proteins (albumin, pre-albumin, transferrin, and retinol binding protein) can be good nutritional indicators in the critically ill patient. They reflect the ability of the liver to incorporate amino acids into protein synthesis, their half-life is not very long, and their concentrations fall rapidly in acute disease and recover in a relatively short time [7,16]. However, no relationship has been found between changes in the levels of these proteins and the nitrogen balance [16,21].

Albumin is a parameter that is widely used in nutritional evaluation due to its high specificity. However it has a low sensitivity as a nutritional marker as it has a long plasma half-life (20 days) and therefore is not a good parameter for monitoring nutritional status due to its low sensitivity to acute changes [4]. Other body proteins with shorter half-lives are better alternatives for evaluation of the protein nutritional status in the critically ill patient.

Pre-albumin, with a short half-life of two days and a small volume of distribution, is very sensitive and specific to changes in the nutritional status. Variations in its concentration can be observed in less than seven days after changes in the diet, and some studies report a good correlation between the levels of this protein and the nitrogen balance [16,21,25–27]. Pre-albumin is a useful parameter for monitoring, re-nutrition and the evolution of nutritional status of the seriously ill patient and is the only valid parameter for evaluation of the nutritional status in renal failure [4].

Transferrin, the iron transport protein, has a short half-life of eight to ten days, and a small volume of distribution. However, its value as a nutritional indicator is lower than that of pre-albumin due to its low sensitivity and specificity when analyzed individually. Its levels are altered in liver disease, iron deficiency anaemia, nephrotic syndrome, and by multiple transfusions or the administration of aminoglycosides and cephalosporins. Even so, in the absence of alterations of iron metabolism, the transferrin concentration is a parameter that provides an adequate evaluation of the protein nutritional status. The rapid increase in transferrin and pre-albumin on starting enteral nutrition supports the hypothesis that when the energy and protein delivery is adequate in the critically ill child, hepatic synthesis of body proteins is rapidly stimulated, leading to a rise in their serum concentrations [16,21,27].

Retinol binding protein has a very short half-life of 12 hours, and its levels fall with malnutrition; however, levels also fall with liver disease, infection, and with intense stress. Retinol binding protein is a good marker of the nutritional status evolution and re-nutrition, but is not of value in patients with renal failure [4].

Fibronectin is synthesized in endothelial cells, hepatocytes, macrophages, and fibroblasts and it has a short half-life of between four and 24 hours. During fasting and in patients with malnutrition, fibronectin levels fall earlier than those of other proteins synthesized in the liver, and increase rapidly after restoration of an adequate energy delivery. It is considered to be a good marker of the nutritional status, although its concentration also falls in numerous acute diseases, such as sepsis and burns, and also in the postoperative period [16,25].

Alterations in electrolyte and mineral levels are common in the critically ill patient, but these changes are not generally secondary to problems of nutrition.

The incidence of hypocalcaemia, hypophosphataemia, and hypomagnesaemia is high in the critically ill child [24]. The most common causes of hypocalcaemia in the critically ill patient are cardiac surgery, sepsis, multiple transfusions, severe pancreatitis, acute renal failure, severe trauma, and multiorgan failure. Hypophosphataemia is usually secondary to fasting, to an increase in anabolism, which increases phosphate consumption, to the intravenous administration of glucose, or to diarrhoea [16,24]. The most common causes of hypomagnesaemia in the critically ill patient are a reduced delivery, the use of loop diuretics, extracorporeal surgery, and diarrhoea. Surgery and infections lead to a fall in the serum concentrations of zinc and iron due to hepatic redistribution and to the binding of iron to ferritin and of zinc to metallothionein. In the case of copper, the levels rise due to an increased synthesis of ceruloplasmin as an acute-phase reactant. However, the most common cause of zinc and copper deficiency is a reduced supply [16,24].

## 6. Repercussions and Complications of Malnutrition in the Critically Ill Child

In the critically ill child, complex metabolic changes occur in order to mobilize the substrates necessary for defense of the body against aggression. Two different phases can be identified in this response of the body to disease: an initial phase of a few hours during which there is a rapid fall in the metabolic rate, with a decrease in oxygen consumption and in energy production; and a second phase, regulated by hormonal changes, during which there is hypercatabolism with a negative nitrogen balance, loss of weight and muscle mass, and altered dynamics of carbohydrate metabolism [8,9,16,21].

These changes lead to protein-energy malnutrition, characterized by a loss of body protein and fat deposits and a fall in the serum protein levels. They are more common in children under two years of age and in more severely ill patients and are associated with an increase in mortality and morbidity [27–29].

## 7. Importance of Nutrition in the Critically Ill Child

One of the fundamental roles of nutrition in the healthy child is to enable growth and development. In contrast, the critically ill child uses nutrients principally to defend the body against disease and, even if a high energy delivery is provided, the body is not able to use this for growth [2]. In these children, nutritional treatment must therefore be orientated to delivering those substrates that favor the maintenance of organ function and recovery from disease [7].

Although nutrition is receiving ever more attention as a part of the treatment of the critically ill child, there are few studies that have systematically analyzed its efficacy [30,31], and recommendations are almost wholly based on expert opinion [2,11,17]. At the present time there are many questions and little evidence regarding the energy delivery and type of nutrients that a critically ill child must receive, the time of starting nutrition, and the most appropriate route for administration and monitoring methods.

A number of studies have analyzed the methods for administering nutrition to the critically ill child. A recent publication reported the results of a survey undertaken in 24 PICUs in 14 Latin-American countries and offers a vision of the way in which nutrition is provided to the critically ill child in Latin-America [32].

It should be noticed the importance of the refeeding syndrome in the critically ill children who can be moved from the starved state to the fed state rapidly via enteral or parenteral nutrition. This syndrome causes severe complications such as electrolyte abnormalities, heart failure, respiratory failure, and death [33].

## 8. What Calorie Delivery Does the Critically Ill Child Require?

The critically ill child typically has a lower energy expenditure than the healthy child due to the reduced motor activity and work of breathing, in addition to the sedation, relaxation, and hypothermia; however these patients have different requirements for certain nutrients [2,6]. Only a small percentage of children, those with persistent high fever, trauma, major burns, or prolonged admission to the PICU, have an increased metabolic rate.

The ideal energy delivery that a critically ill child should receive is not known. An insufficient calorie delivery leads to a loss of organ reserves and of the response capacity of the body to aggression. In contrast, an excessive energy delivery can lead to metabolic overload without stimulating anabolism, with increased carbon dioxide production and prolongation of mechanical ventilation.

Numerous formulae have been used to calculate the energy delivery that the critically ill child requires, though none has been found to be useful [6]. Indirect calorimetry, calculated from analysis of the inspired and expired gases, is the best method for evaluating individual energy expenditure [2,6,34]. Although it would be ideal to measure calorimetry continuously, several studies have demonstrated that a measurement over 30 minutes to two hours provides a reliable estimate of daily energy expenditure. However, indirect calorimetry monitors are expensive and complex to use, and their use has not become widespread; furthermore, they are not reliable in children administered an FiO_2_ greater than 60%, if there are leaks of greater than 10% from the endotracheal tube, or during extracorporeal membrane oxygenation or venovenous continual renal replacement therapy [2,6,34]. Recently, indirect calorimetry modules have been designed that are adapted to the monitors commonly used in the PICU and that enable its use to be extended. Studies using indirect calorimetry have estimated that the energy expenditure of the critically ill child varies between 40 and 65 Kcal/kg/day and thus, if indirect calorimetry is not available, this is the energy delivery that should be used when starting nutrition [2,6,34].

## 9. What Type of Nutrition Should the Critically Ill Child Receive?

For a long time it was thought that severely ill patients could not tolerate enteral nutrition because there would be marked alterations of gastrointestinal motility, digestion, and absorption; parenteral nutrition was therefore considered to be the initial method of feeding.

The principal advantages of parenteral nutrition are that nutrient delivery is independent of the state of the digestive tract, an exact energy delivery can be ensured, and it does not have the risk of digestive tract complications or pulmonary aspiration.

However, numerous studies in the past decade have demonstrated that the majority of critically ill children tolerate enteral nutrition well [2,7,11,16,17,35]. Enteral nutrition is more physiological, has a trophic effect on the intestinal mucosa, and stimulates the intestinal immune system, decreasing bacterial overgrowth and translocation; it therefore reduces the incidence of sepsis and multiorgan failure. Furthermore, enteral nutrition is associated with fewer hepatic and metabolic complications than parenteral nutrition, it is cheaper, it does not require special preparation, and it can be started and modified at any time. At the present time, although very few studies have compared the efficacy and complications of enteral and parenteral nutrition, and although enteral nutrition has not been shown to reduce mortality or length of PICU stay in comparison with parenteral nutrition, the majority of paediatric intensive care physicians consider that enteral nutrition should be the first method for feeding the critically ill child and that it should be started early [2,7,11,16,17,35–37].

Parenteral nutrition should be reserved for those children in whom enteral nutrition is contraindicated or is not tolerated. The contraindications to enteral nutrition have decreased, and now include the following: patients with intestinal obstruction or severe gastrointestinal damage, ischaemia or inflammation; gastrointestinal haemorrhage; peritonitis; and paralytic ileus. In theory, recent gastrointestinal surgery should not be considered a contraindication to enteral nutrition. When it is not possible to achieve an adequate nutrient delivery with enteral nutrition, mixed enteral and parenteral nutrition may be administered [7].

## 10. Gastric or Duodeno-Jejunal Nutrition?

The majority of critically ill children receive gastric enteral nutrition as it is more physiological, simpler, and can be started more quickly [31,32]. In many PICUs, gastric nutrition is administered continuously due to the belief that this facilitates tolerance and reduces the volume of gastric residue and, thus, the risk of aspiration. There are no studies that have compared continuous and intermittent gastric administration in critically ill children, although no differences have been found in the incidence of complications in adults.

Gastric nutrition is occasionally poorly tolerated, particularly by children with more serious illness and on mechanical ventilation with deep sedation. This leads to a situation in which a high percentage of critically ill children with gastric nutrition do not receive the prescribed intake.

Transpyloric duodeno-jejunal nutrition, also known as transpyloric post-pyloric enteral nutrition, reduces the volume of gastric residue and the number of interruptions, enabling the nutrition to be increased more rapidly in order to reach the prescribed volume [38,39]. In addition, in the critically ill child and, in particular, in the infant, the introduction of the transpyloric tube is simple and nutrition can therefore be started early. Transpyloric enteral nutrition is well tolerated and has few complications, although it has not been shown to reduce the incidence of pulmonary aspiration [2,7,38,39]. There is only one study in critically ill children that has compared gastric and transpyloric nutrition [30]. Children with transpyloric enteral nutrition reached a higher calorie delivery with the same incidence of complications. In premature neonates, by contrast, transpyloric enteral nutrition has not been shown to have any benefit over gastric feeding and it can increase the incidence of diarrhoea or food intolerance [40].

## 11. What Type of Nutrients should the Critically Ill Child Receive?

There are no studies that have analyzed the ideal diet in the critically ill child and there are no new products specifically designed for these patients. For patients of one to two years of age, many authors administer maternal milk or infant formula due to their availability, digestibility, excellent tolerance, and low osmolarity; complete enteral diets are used in older children. An enteral diet for ill infants has recently come onto the market, though studies are necessary to analyze its efficacy in critically ill children and to compare it with infant formula. Nor are there any studies that have demonstrated the advantages of hydrolyzed proteins over complete diets [7].

There is a pronounced increase in catabolism in critically ill patients, which makes it difficult to achieve a positive nitrogen balance. The protein delivery typically recommended in the critically ill child is of 2.5–3 g/kg/day. Some studies have found that a hyperproteic diet increases protein synthesis and improves the nitrogen balance, although it does not reduce catabolism or improve the prognosis [7,21].

The terms immunonutrition and pharmaconutrition have become widespread in recent years. These terms refer to the concept that nutrition serves not only for the administration of energy substrates but also of other substances that are deficient in the critically ill patient and/or that modify the inflammatory and immune responses. Immunomodulating diets supplemented with glutamine, arginine, ribonucleic acid, antioxidants, and omega-3 fatty acids have been used in critically ill adults, with very variable clinical results; some authors have reported a reduction in the incidence of nosocomial infection and even in mortality, whereas others have reported negative effects [41]. Although there are no specific immunomodulating diets for children, one study found a higher incidence of diarrhoea after administering an immunomodulating diet designed for adults to critically ill children [42].

## 12. When Should Nutrition be Started?

Critically ill children are particularly vulnerable to the effects of fasting and of prolonged stress, as they have lower percentages of muscle and fat and higher basal energy requirements than adults. A number of studies have demonstrated that the majority of critically ill children tolerate early enteral nutrition well, with no increase in the incidence of complications [2,39]. It would therefore appear reasonable to recommend that critically ill children are not kept fasting for more than 24 to 48 hours. However, many critically ill children start nutrition very late and do not receive the prescribed energy delivery, usually because of the need for fluid restriction, interruptions due to procedures, poor tolerance, or mechanical problems (the tube becomes obstructed or falls out). A recent study found that although 93% of critically ill children received nutrition on the third day after admission, the prescribed energy delivery was only achieved on the fifth day [43].

## 13. Characteristics of Parenteral Nutrition

The greatest advances in parenteral nutrition in recent years concern the lipids. The lipid content of parenteral nutrition reduces gluconeogenesis and fat deposition and stimulates lipid oxidation and protein retention. It is interesting that, although many studies have demonstrated the compatibility of lipids with the other substrates of parenteral nutrition, and that combined administration reduces the number of intravenous catheters and manipulations required, reducing the risk of infection, there are still many PICUs that administer lipids separately [32]. New lipid preparations for parenteral nutrition have been developed in recent years, with increased omega-3 and omega-6 fatty acid content based on soya oil, fish oil, and olive oil. These products are metabolized better and have been shown to have anti-inflammatory and immunomodulatory effects. However there are no studies that have analyzed their effects in the critically ill child [7].

A recent retrospective study showed that parenteral nutrition with commercial solutions in critically ill children reduced the cost with respect to preparation of the solutions in the pharmacy department; however, that study did not compare safety, contamination and adverse effects of the preparations [44].

## 14. How Should Nutrition be Monitored in the Critically Ill Child

The objectives of monitoring nutrition in the critically ill child must be to evaluate its effect on the nutritional status and to detect adverse effects. Nutritional evaluation in the critically ill child is complicated because, as has been stated above, anthropometric methods (weight and skinfold thickness), which are the simplest, are relatively insensitive to short-term changes and can be affected by variations in body composition (oedema or dehydration). Some serum proteins with a short half-life, such as pre-albumin and retinol binding protein, are relatively sensitive and specific indicators of the nutritional status, although there is still little experience with their use in critically ill children. Indirect calorimetry is the best method for measuring energy expenditure and, together with the nitrogen balance, which is easy to calculate and allows protein catabolism to be determined, and analysis of the nutrition administered, the balance between the delivery and utilization of energy and immediate substrates can be calculated.

The greatest limitation to achieving adequate enteral nutrition in critically ill children is the onset of gastrointestinal complications (38). The most common gastrointestinal complications are abdominal distension, excessive gastric residues, vomiting, diarrhoea, and constipation [31,38]. In one study, the incidence of complications of enteral nutrition was the following: vomiting, 17.9%; abdominal distension, 13.2%; excessive gastric residues, 4.7%; and diarrhoea, 11.3% [31]. In our experience, the incidence of gastrointestinal complications in 526 critically ill children receiving transpyloric enteral nutrition was 11.5%, though definitive withdrawal of the nutrition was required in only 2.1% of patients [38]. The most important risk factors for gastrointestinal complications are shock, acute renal failure, hypophosphataemia, and the administration of catecholamines, sedatives, and muscle relaxants. There are no clinical studies that have evaluated the usefulness of special diets, prokinetics, and laxatives in the prevention and treatment of these complications in the critically ill child.

## 15. Conclusions

Malnutrition is very common in the critically ill child and has a negative effect on prognosis, increasing the incidence of complications and the morbidity and mortality. Although scientific evidence is still scarce, it would appear reasonable to start enteral nutrition early in the critically ill child, within the first 24 to 48 hours after admission, when oral feeding is not possible. Energy delivery must be adjusted to energy expenditure and, if this is not possible, a delivery of 40–65 kcal/100calories metabolized/day should be administered, with a protein delivery of 2.5–3 g/kg/day, reaching the prescribed energy delivery within the first 48 to 72 hours. Gastric feeding can be the initial method of nutrition in the majority of critically ill children, keeping transpyloric feeding for more seriously ill patients on mechanical ventilation and who are receiving high doses of sedatives and muscle relaxants, those who have a higher risk of pulmonary aspiration, and those who do not tolerate gastric feeding or in whom an adequate energy delivery cannot be achieved. Parental nutrition should only be used when enteral nutrition is contraindicated or is not tolerated. Frequent monitoring of nutritional and metabolic parameters should be performed, together with vigilance for complications.

The objectives in the future should be to administer individualized nutrition in accordance with the specific characteristics of each critically ill child and to be able to adjust the nutrition continuously according to the metabolic changes and nutritional status. For this, it is important to conduct studies to identify sensitive methods for the evaluation of nutrition that can be applied in the majority of critically ill children. In addition, prospective clinical trials are needed to compare continuous *versus* intermittent gastric feeding, and to analyze the usefulness of the diet guided by calorimetry and hyperproteic, hydrolyzed, and immunomodulating diets, and the effectiveness of prokinetics and laxatives for the prevention and treatment of the increased incidence of constipation.
